# After the fire: Post-pandemic reflections

**DOI:** 10.1177/1751143720937957

**Published:** 2020-07-02

**Authors:** Daniel Martin

**Affiliations:** 1 Intensive Care Unit, Royal Free Hospital, Pond Street, London, UK; 2 Peninsula Medical School, University of Plymouth, Plymouth, UK

Over the space of a few months, virtually everything we know has been turned on its head. In Wuhan (China), during the closing months of 2019, a spillover event occurred in which the severe acute respiratory syndrome coronavirus 2 (SARS-CoV-2) was transferred from its animal host population into humans.^1,2^ Spread mainly by respiratory droplets, the virus has a basic reproduction number (R_0_) of between 2 and 6 in an unprotected population, making it more contagious than influenza, but considerably less so than measles. As SARS-CoV-2 rapidly spread across the planet, aided by our love of intercontinental travel, the local market outbreak of a mystery respiratory illness became a global pandemic of unimaginable proportions.^3^ Coronavirus disease 2019 (COVID-19), the illness that occurs in humans as a result of infection with SARS-CoV-2, has dominated the news for most of 2020 to date, and transformed the way in which we live, work, socialise, shop, exercise and communicate. In addition to this, it has placed intensive care medicine at the very centre of healthcare planning in the future, as the pandemic highlighted the weakness in our local, regional and national systems for dealing with crisis of this magnitude. The herculean efforts seen in intensive care units (ICUs) around the world are a testament to the dedication of those in our specialty and those who came to support us in our hour of need. However, for many of us, the situation was (and may still be) overwhelming and unsustainable; none of us would be eager to return to it in a hurry. Once we have all taken a well-earned break, we need to brace ourselves for whatever lies ahead, equipped with the knowledge and experiences gained during this pandemic.

## The disease

Much has been written about COVID-19 in the last few months, both in medical and scientific journals and in the press. One of the many challenges for front-line healthcare workers during the pandemic was wading through the abundance of daily literature updates and commentaries to try to distinguish fact from fiction. Inboxes were overwhelmed with hot-off-the-press articles, which ranged from case reports through to randomised controlled trials, alongside more leftfield hypotheses and ‘viral’ (using the internet rather than infections definition) emails. In non-pandemic times, we would never dream of listening to the musings of a lone voice on something as important as the management of critically ill patients. However, during times of extreme pressure, the desire for new information can overwhelm our usual caution with regard to new evidence, and encourage us to let our guard down and abandon the checks and measures that we would normally use to critically appraise it. Usually it takes years, if not decades, for us to carefully navigate a robust pathway from the discovery of a disease to the development of successful treatments for it. Those of us working in intensive care are sadly familiar with the years of failed searches for the ‘magic bullet’ to treat syndromes such as sepsis or acute respiratory distress syndrome (ARDS), so it would seem incredibly unlikely that we would stumble across one for this brand-new disease in a matter of mere months. We have grown to accept the fact that almost every large-scale multi-centre RCT of a new (or old) intervention conducted in intensive care leads to a frustratingly neutral finding. In the UK, the speed at which the research community turned its attention to tackling COVID-19 was extraordinary. The entire process of study approval was streamlined to allow crucial high-priority studies to begin recruiting during the pandemic. Nationwide studies to capture data from COVID-19-positive patients were immediately activated, and as a result, we now have some of the most comprehensive datasets in the world.^4,5^ Early acquisition of data that is regularly updated is arguably our most effective tool in the fight against an emerging disease. Francis Bacon's iconic phrase from the end of the 16th century, ‘*scientia potentia est*’ (knowledge is power), was one that resonated throughout the pandemic. As time progresses, it will be interesting to see which of the potential treatments that were evaluated in early trials will emerge as effective and how these may specifically benefit critically ill patients.

In a way that rarely occurs in modern medicine, we also learnt a great deal about this disease from ‘trial and error’. Back in March 2020 when the first cases started to be admitted to ICUs in the UK, we were convinced we knew what we were dealing with. We expected patients to arrive on ICU with ARDS in the guise of ‘single organ failure’ and geared up to manage it as we have learnt to over the last few decades.^6^ Whilst not entirely incorrect in our assumptions, we did need to modify our tactics a little to account for COVID-19's idiosyncrasies. In April 2020, the Intensive Care Society produced a synthesis of clinical experience from UK intensivists that summarised the state of play at that time.^7^ Key themes within the document were: the effectiveness of proning for severe hypoxaemia; the potential harm from excessive positive end-expiratory pressure (PEEP); the high incidence of iatrogenic renal failure from excessive diuresis; and the high rate of re-intubation. Releases like this provided support to clinicians who were likely to have made similar observations in their own ICU and facilitated large-scale adaptation to a rapidly developing landscape.

## The response

I can vividly recall the first patient with COVID-19 being admitted to my own ICU. Despite being prepared for this eventuality, there was considerable apprehension amongst staff, with the realisation that the outbreak had begun. We had prepared our ICU to receive up to two patients, and if there was no longer any capacity within the NHS England Airborne High Consequence Infectious Diseases network, we would expand that to four.^8^ But this would be our absolute maximum. At the peak of the pandemic, we were caring for more than 60 COVID-19-positive critically ill patients at once, spread across three floors of the hospital, having commandeered the operating theatres, recovery areas and an outpatient treatment area. Each day, changes were occurring to the structure and processes of the hospital that would usually take several committees a number of years to even contemplate. In short, the response to this pandemic in hospitals throughout the National Health Service in the United Kingdom and around the world was nothing short of miraculous. As the admissions began to increase, units adapted within the boundaries of their walls, using side rooms to isolate infected individuals and then switching the model to cohorted bays of COVID-19-positive patients. Once full, ICUs expanded outwards, commonly using operating theatre complexes as an overflow area, as the necessary equipment and facilities to manage mechanically ventilated patients was already available there. In order to staff these expanded ICUs, we drafted in personnel from within and outside of our hospitals. Perhaps one of the most incredible local stories was a group of diver medical technicians volunteering to help on our ICU.^9^ Training had to occur for those unfamiliar with ICU to enable them to perform specific roles such as nursing support, proning patients, intravascular access, data collection, equipment management and the extremely sensitive and critical task of communicating with the families that, for the first time in our history, were not permitted to enter the hospital. The barrier we had to place between patients and their loved ones was necessary to protect healthcare workers and the public but created unthinkable scenarios, particularly at the end of life. Teams worked around the clock to communicate to families and used video technology to bridge this emotional gulf. Staff rose to the challenges and many grew into new roles. Our ICU family expanded beyond our wildest dreams; shift handovers became a hospital-wide event where staff from all manner of backgrounds would congregate in a safe manner to keep ICU running effectively. Support arrived in abundance from outside the hospital, in the form of food, clothing, accommodation, essential supplies and all sorts of treats. Everyone wanted to play their part in the greatest challenge ever faced by the modern healthcare system.

## The aftermath

Every story comes to an end and, at the time of writing, the peak of the pandemic in the United Kingdom appears to have passed and normality is slowly beginning to return. De-escalation is as logistically perplexing as escalation and hospitals have had to make decisions on when and how their huge ICU teams should begin to stand down to allow a shrinkage of their floorspace, back to their original setup. As the number of admissions through emergency departments began to plateau, then fall, the pressure on wards and ICUs stabilised. In London, we are now in a state of steady decline in terms of number of inpatients, and an there is a growing eagerness to resume services such as elective surgery. The de-escalation has been deliberately cautious in many centres due to the underlying fear of a ‘second wave’ as was seen in the influenza pandemic of 1918.^10^ Whilst in 1918, the second wave was unlikely have been due to any premature easing of social isolation, there is good reason to believe that if we were all to abandon the practices we have all become so familiar with (quarantine, isolation, shielding, social distancing, the wearing of masks and the washing of hands) a second wave may have already begun by the time this article is published. So, whilst we may want to celebrate and get back to whatever we were doing before the pandemic, it is important to remember that ‘*it ain't over 'til it's over*’. The problem is, how do we know when it is over? How long do we wait until we can be sure that COVID-19 is gone for good? Can we ever be sure of that? Do we need to remain on guard until a vaccination programme is established? So many vital questions that no one can yet answer with any authority.

## The future

We should not believe the media's myth that pandemics are a 100-year phenomenon. The frequency of pandemics has been increasing over the last few decades, particularly due to strains of influenza (H2N2 (‘Asian flu’) in 1957–1958, H3N2 in 1968, and H1N1 (‘swine flu’) in 2009–2010), and is likely to continue doing so because of the intermingling of human civilisation and environments close to animal hosts of disease.^11^ As healthcare workers responsible for the survival of critically ill patients, we need to ensure we are ready for the next pandemic, and the one that follows it. Many useful articles are now appearing in the literature to help us think this through in a much calmer way than we were perhaps capable of in the heat of the moment during our first pandemic.^12^ Crucial to our future response will be linking local ICUs to provide a more streamlined service to the population, and developing a common language and approach to aid escalation as a pandemic develops. The CRITCON framework is an excellent example of this, outlining an escalation plan and operational matrix for hospitals to follow during a developing pandemic.^13^ Strategic planning is also necessary to minimise the impact of future pandemic surges to the delivery of other essential medical services. Particular attention should be devoted to mitigating the secondary harm from the reduction in urgent elective surgery (particularly for cancer) during the surge.^14^ Whilst we cannot be experts in every facet of pandemic medicine, most of us have gained a vast amount of hard-won experience in this first phase, and must find ourselves in far better place than at the beginning of 2020 with regard to preparedness for what may lie ahead of us.

## Conclusion

Thank you to everyone who played a role in what has likely been the largest ever global healthcare response to an infectious disease. As a specialty, we have demonstrated adaptability to a rapidly developing novel disease and global catastrophe, and shone a light on the absolute necessity of intensive care medicine in a modern healthcare system.
